# Thyrotoxic Periodic Paralysis as an Initial Manifestation of Hyperthyroidism in a Young Male Patient

**DOI:** 10.7759/cureus.116871

**Published:** 2026-09-24

**Authors:** Casandra Valadez Villalobos

**Affiliations:** 1 Internal Medicine, Universidad Nacional Autónoma de México, México City, MEX

**Keywords:** acute flaccid paralysis, graves disease, hypokalemia, thyrotoxicosis, thyrotoxic periodic paralysis

## Abstract

Thyrotoxic periodic paralysis (TPP) is a rare but potentially fatal complication of hyperthyroidism, characterized by acute muscle weakness and hypokalemia. Although most frequently described in Asian populations, it can occur in other ethnic groups and is often underdiagnosed. We present the case of a 21-year-old Mexican man with acute lower-extremity weakness.

Initial laboratory evaluation revealed a serum potassium level of 2.7 mEq/L and biochemical thyrotoxicosis. The patient received 60 mEq of intravenous potassium chloride over six hours, with complete recovery of muscle strength approximately eight hours after treatment initiation, supporting the diagnosis of TPP. Thyroid scintigraphy was suggestive of diffuse toxic goiter, while the elevated thyrotropin receptor antibody (TRAb) level supported Graves' disease as the underlying etiology. This case highlights the importance of considering TPP in the differential diagnosis of acute flaccid paralysis and emphasizes the need for early diagnosis and treatment to prevent potentially serious complications.

## Introduction

Thyrotoxic periodic paralysis (TPP) is a rare but potentially serious complication of hyperthyroidism, characterized by acute episodes of flaccid muscle weakness and hypokalemia in the context of thyrotoxicosis.

Although TPP has been reported more frequently in Asian populations, its incidence among North American patients with hyperthyroidism has been estimated at 0.1-0.2%, approximately one-tenth of that reported in Asian populations [1]. It can also occur in other ethnic groups and may remain underdiagnosed. Despite the higher prevalence of hyperthyroidism in women, TPP occurs predominantly in young men [1,2].

The pathophysiology of TPP is primarily related to an intracellular shift of potassium, rather than a total body loss of potassium. This phenomenon is mediated by increased Na⁺/K⁺-ATPase activity induced by excess thyroid hormones and heightened beta-adrenergic sensitivity. Genetic susceptibility may also contribute to TPP, including variants in KCNJ18, which encodes the skeletal muscle potassium channel Kir2.6 [2]. Episodes may be precipitated by factors such as high-carbohydrate meals, alcohol intake, strenuous exercise, or rest following exercise [1,2]. Graves' disease is the most frequently associated cause of this condition [3,4]. However, TPP has also been reported with toxic nodular disease, thyroiditis, and iodine- or drug-induced thyrotoxicosis [4,5].

Early recognition of TPP is crucial, as it can manifest as acute flaccid weakness and be associated with severe electrolyte imbalances and potentially fatal cardiac complications [4]. Therefore, TPP should be considered in the differential diagnosis of acute flaccid paralysis, particularly in young men with hypokalemia and clinical or biochemical manifestations of hyperthyroidism.

In patients presenting with acute flaccid paralysis and hypokalemia, the differential diagnosis includes familial or sporadic hypokalemic periodic paralysis, renal potassium-wasting disorders, and neurological conditions such as Guillain-Barré syndrome. The presence of thyrotoxicosis favors TPP, whereas a family history may suggest familial hypokalemic periodic paralysis. Urinary potassium measurements and acid-base status can help assess renal potassium handling. Blood pressure may raise suspicion for primary aldosteronism, while medication history can identify diuretic-related hypokalemia. Neurological examination, particularly the assessment of sensation and deep tendon reflexes, helps distinguish TPP from primary neurological disorders [4].

## Case presentation

In October 2023 (Day 1), a 21-year-old Mexican man with no relevant medical history presented to the emergency department with progressive lower-extremity weakness. The patient reported an involuntary weight loss of approximately 5 kg over the previous two months, generalized weakness, tremor, palpitations, anxiety, and heat intolerance.

No clear precipitating factor was identified before the episode of weakness. The patient denied a recent high-carbohydrate meal, strenuous physical activity, acute alcohol intake, or a specific acute stressor preceding the attack. Although the patient reported occasional alcohol consumption, no temporal relationship with the episode was identified. Diuretic use and glucocorticoid exposure before symptom onset were denied.

On presentation that day, he experienced an acute worsening of lower-extremity weakness, which made walking difficult. Initial vital signs revealed a blood pressure of 156/76 mmHg, a heart rate of 106 beats/minute, a respiratory rate of 20 breaths/minute, a temperature of 36.8 °C, and an oxygen saturation of 93% on room air. On subsequent assessment, oxygen saturation was 95% on room air. Pulmonary examination showed preserved breath sounds and adequate air entry. Neurological examination showed symmetrical proximal weakness confined to the lower extremities, with muscle strength graded as 1/5, while upper-extremity strength was preserved. Deep tendon reflexes were 2+ and symmetric bilaterally. Sensory examination and mental status were intact. A neurology consultation was obtained, and other neurological causes of his presentation were ruled out clinically. An electrocardiogram (ECG) showed sinus tachycardia at 105 beats/minute, with a QT interval of 280 ms and a corrected QT (QTc) of approximately 370 ms. No prominent U waves, ectopic beats, or other acute electrocardiographic abnormalities were identified. 

Serum potassium measured at emergency department presentation on Day 1 was 2.7 mEq/L (Table 1). Given the accompanying symptoms suggestive of thyrotoxicosis, thyroid function tests were subsequently obtained on Day 3. These demonstrated suppressed thyroid-stimulating hormone and elevated free triiodothyronine and free thyroxine levels, confirming biochemical thyrotoxicosis. Blood gas analysis obtained on Day 3, after the initial potassium repletion, showed a mild metabolic acidosis (pH 7.39, pCO₂ 32 mmHg, and HCO₃⁻ 19.4 mmol/L) with a lower-than-expected pCO₂, suggesting a concurrent respiratory alkalosis. The calculated serum anion gap was 14.6 mEq/L.

**Table 1:** Initial and serial laboratory findings during the clinical course Initial and serial laboratory findings demonstrating biochemical thyrotoxicosis, the evolution of serum potassium levels, additional biochemical evaluation, and the subsequent TRAb result. Reference ranges correspond to the institutional laboratory when available. Standard adult reference ranges were used when institutional ranges were unavailable.

Laboratory parameter	Date/clinical time point	Patient result	Reference range
Potassium	Day 1, before initial potassium replacement	2.7 mEq/L	3.5-5.1 mEq/L
Blood gas pH	Day 3	7.39	7.35-7.45
pCO₂	Day 3	32 mmHg	35-45 mmHg
HCO₃⁻	Day 3	19.4 mmol/L	22-26 mmol/L
Serum sodium	Day 3	140 mEq/L	135-145 mEq/L
Serum chloride	Day 3	106 mEq/L	98-107 mEq/L
Calculated serum anion gap	Day 3	14.6 mEq/L	Not applicable (calculated value)
TSH	Day 3	0.0008 µIU/mL	0.465-4.68 µIU/mL
Free T3	Day 3	14.84 pg/mL	2.77-5.27 pg/mL
Free T4	Day 3	2.32 ng/dL	0.78-2.19 ng/dL
Potassium	Day 4, after initial replacement	4.1 mEq/L	3.5-5.1 mEq/L
Potassium	Day 8	3.3 mEq/L	3.5-5.1 mEq/L
Magnesium	Day 8	2.0 mg/dL	1.80-2.40 mg/dL
Phosphate	Day 8	4.1 mg/dL	2.5-4.5 mg/dL
Potassium	Day 10	4.5 mEq/L	3.5-5.1 mEq/L
24-hour urine volume	Days 10-11, collected after potassium repletion	1.8 L	Not applicable
24-hour urinary sodium	Days 10-11, collected after potassium repletion	190 mmol/24 h (190 mEq/24 h)	40-220 mmol/24 h
24-hour urinary potassium	Days 10-11, collected after potassium repletion	120 mmol/24 h (120 mEq/24 h)	25-125 mmol/24 h
24-hour urinary chloride	Days 10-11, collected after potassium repletion	200 mmol/24 h (200 mEq/24 h)	110-250 mmol/24 h
Urine pH	Days 10-11, collected after potassium repletion	6.0	4.6-8.0
Calculated urine anion gap	Days 10-11, collected after potassium repletion	+61.1 mEq/L	Not applicable (calculated value)
Thyrotropin receptor antibody (TRAb)	12 days after hospital discharge	4 IU/L	Positive cutoff: >1.5 IU/L

Intravenous potassium replacement was initiated on Day 1 with 60 mEq of potassium chloride administered over six hours at a rate of 10 mEq/hour. Approximately eight hours after treatment initiation, the lower-extremity weakness resolved completely, with muscle strength returning to 5/5; upper-extremity strength remained preserved. Serial serum potassium measurements are presented in Table 1. Serum potassium increased from 2.7 mEq/L at presentation to 4.1 mEq/L on Day 4, after the initial correction. 

Based on the combination of acute muscle weakness, hypokalemia, biochemical thyrotoxicosis, and the clinical response to potassium replacement, the diagnosis of thyrotoxic periodic paralysis was clinically supported. The patient was admitted to the Internal Medicine service on Day 6. Methimazole 15 mg orally every 12 hours was initiated on Day 6.

On Day 8, hypokalemia subsequently recurred, with a serum potassium level of 3.3 mEq/L. An additional infusion of 40 mEq of intravenous potassium chloride was administered over four hours at a rate of 10 mEq/hour. Propranolol 20 mg orally every six hours was added on Day 8 for treatment of thyrotoxicosis. The total documented cumulative potassium chloride dose during hospitalization was 100 mEq. Serum potassium subsequently increased to 4.5 mEq/L on Day 10. No rebound hyperkalemia was documented during hospitalization.

Serial potassium measurements and additional investigations, including serum magnesium, phosphate, and acid-base assessment, were obtained (Table 1). A 24-hour urine collection for urinary electrolyte assessment was initiated on Day 10, after potassium repletion, and completed on Day 11. Urinary potassium was 120 mmol/24 h (institutional reference range: 25-125 mmol/24 h). Urine pH was 6.0, and the calculated urine anion gap was +61.1 mEq/L. Renal function remained preserved, serum magnesium was normal, and no metabolic alkalosis was present. The patient denied taking diuretics or other medications associated with potassium loss. A subsequent blood pressure measurement was 128/60 mmHg. No family history of similar episodes was reported. 

A chronological summary of the patient's clinical course, diagnostic evaluation, therapeutic interventions, and outcomes is presented in Table 2.

**Table 2:** Clinical timeline of the case Chronological summary of the clinical presentation, diagnostic evaluation, therapeutic interventions, and outcomes during the episode of care.

Date/clinical time point	Clinical event	Findings/intervention	Outcome
Approximately two months before presentation	Onset of thyrotoxic symptoms	Progressive weight loss, tremor, palpitations, anxiety, and heat intolerance	Symptoms persisted
Day 1	Acute neurological presentation	Marked symmetrical proximal lower-extremity weakness developed, with muscle strength graded as 1/5 and difficulty walking	Emergency evaluation and neurology consultation were initiated
Day 1	Hypokalemia documented and initial potassium replacement	Serum potassium was 2.7 mEq/L; intravenous potassium chloride replacement was initiated with 60 mEq administered over six hours at a rate of 10 mEq/hour	Progressive improvement in muscle strength
Day 1, approximately eight hours after treatment initiation	Neurological reassessment	Lower-extremity strength returned to 5/5; upper-extremity strength remained preserved	Complete resolution of weakness
Day 3	Thyroid function assessment	Suppressed TSH and elevated free T3 and free T4 levels demonstrated biochemical thyrotoxicosis	Hyperthyroidism was confirmed
Day 4	Laboratory reassessment	Serum potassium was 4.1 mEq/L	Potassium normalized after initial replacement
Day 6	Internal Medicine admission and antithyroid treatment	Muscle strength was 5/5 with preserved sensation; methimazole 15 mg orally every 12 hours was initiated	Neurological improvement was maintained, and treatment of thyrotoxicosis was initiated
Day 8	Recurrent hypokalemia	Serum potassium decreased to 3.3 mEq/L; an additional infusion of 40 mEq of intravenous potassium chloride was administered over four hours at a rate of 10 mEq/hour	Additional potassium correction was initiated
Day 8	Beta-blocker therapy	Propranolol 20 mg orally every six hours was initiated	Treatment of the thyrotoxic state continued
Day 10	Laboratory follow-up	Serum potassium was 4.5 mEq/L	Potassium was within the reference range; no rebound hyperkalemia was documented
Day 14	Hospital discharge	The patient remained clinically stable without recurrent weakness	He was discharged after an eight-day Internal Medicine stay with outpatient endocrinology follow-up
Day 20 (six days after hospital discharge)	Preparation for thyroid scintigraphy	Methimazole was withheld seven days before thyroid scintigraphy	Antithyroid therapy was temporarily withheld before imaging
Day 26 (12 days after hospital discharge)	Etiologic evaluation of thyrotoxicosis	TRAb was 4 IU/L	The result supported Graves' disease as the underlying etiology
Day 27 (13 days after hospital discharge)	Thyroid scintigraphy	A total of 10 mCi (370 MBq) of intravenous 99mTc-pertechnetate was administered; images were acquired 20 minutes later and demonstrated increased radiotracer concentration with heterogeneous distribution and no focal areas of decreased or increased uptake	The nuclear medicine interpretation was suggestive of diffuse toxic goiter
Within the first year and through approximately three years after hospitalization	Outpatient endocrinology follow-up	Methimazole was continued during outpatient follow-up, and the patient subsequently underwent radioactive iodine therapy. Euthyroidism was documented within the first year.	No recurrent episodes of TPP were documented during approximately three years of follow-up

After remaining hemodynamically stable without recurrence of weakness, the patient was discharged on Day 14, with outpatient endocrinology follow-up. Subsequent outpatient etiologic evaluation included TRAb testing and thyroid scintigraphy. Methimazole had been withheld for seven days before the thyroid scintigraphy. TRAb was elevated at 4 IU/L (positive cutoff: >1.5 IU/L), supporting Graves' disease as the underlying etiology of the thyrotoxicosis. Thyroid scintigraphy was performed using 10 mCi (370 MBq) of intravenous 99mTc-pertechnetate, with images acquired 20 minutes after radiotracer administration. The study demonstrated increased radiotracer uptake with a heterogeneous distribution and no focal areas of decreased or increased uptake. These findings, shown in Figure 1, were suggestive of diffuse toxic goiter but inconclusive for establishing the etiology of thyrotoxicosis.

**Figure 1:**
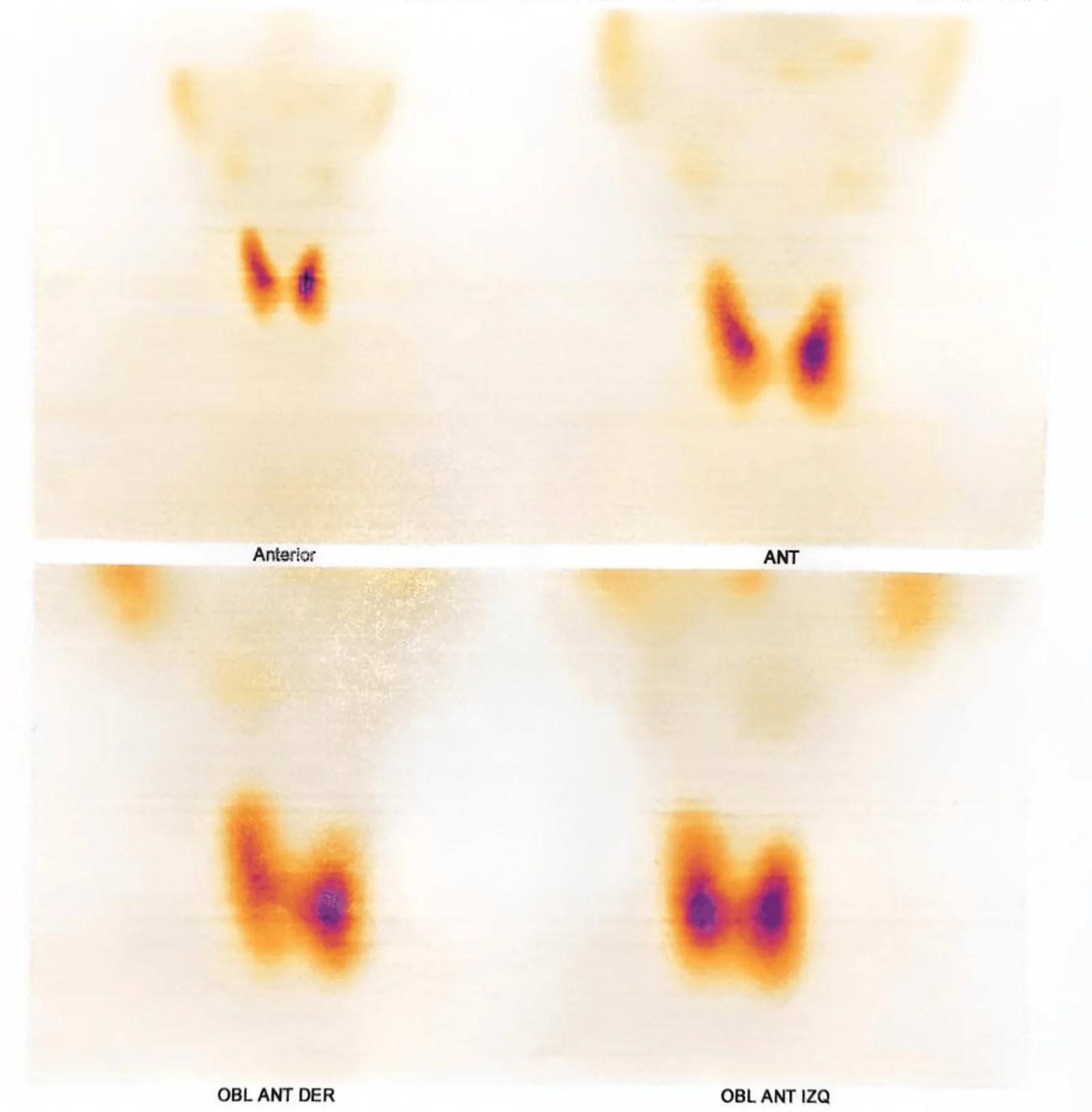
Thyroid scintigraphy Thyroid scintigraphy was performed with 10 mCi (370 MBq) of intravenous 99mTc-pertechnetate. Static cervical images were acquired 20 minutes after injection in anterior and anterior oblique projections, as well as an anterior panoramic view. The thyroid was in its usual anatomical location, with preserved size and morphology, increased radiotracer uptake, and heterogeneous distribution, without focal areas of decreased or increased uptake. Quantitative thyroid uptake was not reported. The scintigraphic findings were suggestive of diffuse toxic goiter but were considered inconclusive for establishing the etiology of thyrotoxicosis.

Outpatient endocrinology follow-up continued for approximately three years after hospitalization. Methimazole was continued during follow-up, and the patient subsequently underwent radioactive iodine therapy. Euthyroidism was documented within the first year. No recurrent episodes of thyrotoxic periodic paralysis were documented during this period.

## Discussion

TPP is a rare but potentially fatal complication of hyperthyroidism. It is classically defined by the triad of thyrotoxicosis, hypokalemia, and acute painless muscle weakness [6,7]. TPP shows a marked male predominance, particularly among young adults, with reported male-to-female ratios ranging from 17:1 to 76:1, despite thyrotoxicosis being more common in women [8]. In North American patients with hyperthyroidism, its incidence has been estimated at 0.1-0.2%, approximately one tenth of that reported in Asian populations [1]. Cases of TPP have also been reported in Latin American populations, including Mexico [9,10]. This case adds to the existing Latin American literature on TPP and further illustrates that the diagnosis should be considered in patients with acute muscle weakness, hypokalemia, and thyrotoxicosis regardless of ethnicity.

The pathophysiology of TPP is primarily related to an intracellular shift of potassium, rather than a total body depletion of this electrolyte. Excess thyroid hormones increase Na⁺/K⁺-ATPase activity in skeletal muscle and enhance beta-adrenergic responsiveness, thereby promoting intracellular potassium influx [2,6]. The resulting hypokalemia alters muscle membrane excitability and manifests clinically as acute flaccid weakness [10].

Clinically, TPP typically presents with sudden, symmetrical, and proximal muscle weakness, predominantly affecting the lower extremities. Sensation and mental status are usually preserved, which helps distinguish it from other causes of acute neuromuscular weakness [11].

The differential diagnosis of acute flaccid paralysis includes neurological disorders such as Guillain-Barré syndrome, transverse myelitis, poliomyelitis, and myasthenia gravis, as well as metabolic causes such as hypokalemic periodic paralysis. Clinical examination, particularly the distribution of weakness, sensory findings, and deep tendon reflexes, can help distinguish these disorders from TPP [4,11].

The patient presented with a typical clinical pattern, characterized by bilateral proximal weakness of the lower extremities, preserved sensation and mental status, moderate hypokalemia, and biochemical thyrotoxicosis. Complete recovery of lower-extremity muscle strength approximately eight hours after treatment initiation further supported the diagnosis.

Other causes of hypokalemic paralysis were also considered in this patient. Familial or sporadic hypokalemic periodic paralysis was considered less likely because there was no family history of similar episodes and the weakness developed in the setting of biochemical thyrotoxicosis. Renal potassium handling was assessed using a 24-hour urine collection initiated on Day 10, after potassium repletion, and completed on Day 11. Urinary potassium was 120 mmol/24 h. Because the collection was obtained after potassium repletion, this result could not reliably assess renal potassium conservation during the initial hypokalemic episode and was not interpreted as excluding renal potassium loss. Blood gas analysis showed a mild metabolic acidosis (pH 7.39, pCO₂ 32 mmHg, and HCO₃⁻ 19.4 mmol/L) with a lower-than-expected pCO₂, suggesting a concurrent respiratory alkalosis. The calculated serum anion gap was 14.6 mEq/L. Preserved renal function and a normal serum magnesium level, together with the absence of metabolic alkalosis on acid-base assessment, made Gitelman syndrome less likely. Urine pH was 6.0, and the calculated urine anion gap was +61.1 mEq/L. However, because these urinary studies were obtained after potassium repletion and were not contemporaneous with the initial acid-base abnormality, they could not reliably assess urinary acidification during that episode. Therefore, distal renal tubular acidosis was not excluded by these data. Primary aldosteronism was considered unlikely because the patient did not have persistent hypertension, with a subsequent blood pressure of 128/60 mmHg. Diuretic-related hypokalemia was also unlikely because he denied the use of diuretics or other medications associated with potassium loss. Finally, a primary neurological disorder became less likely because sensation and mental status remained preserved, no focal neurological deficits were identified, and muscle strength recovered completely after potassium replacement. Taken together, these findings supported TPP as the most likely cause of the acute weakness.

Graves' disease is the most frequently reported underlying cause of thyrotoxicosis in association with TPP [6,7]. In this patient, TRAb was elevated at 4 IU/L (positive cutoff: >1.5 IU/L). Thyroid scintigraphy demonstrated increased radiotracer uptake with heterogeneous distribution and no focal abnormalities and was interpreted as suggestive of diffuse toxic goiter. Methimazole had been withheld for seven days before scintigraphy; however, the potential influence of prior antithyroid therapy on the uptake pattern cannot be completely excluded. Therefore, scintigraphy alone was considered inconclusive for establishing the etiology of thyrotoxicosis. The positive TRAb result, together with the overall clinical and biochemical findings, supported Graves' disease as the underlying etiology. 

Early recognition of TPP is essential, as severe hypokalemia can be associated with potentially fatal complications, primarily cardiac arrhythmias [2]. Acute management requires cautious potassium replacement because total body potassium is not depleted and excessive supplementation may cause rebound hyperkalemia. Rebound hyperkalemia has been reported more frequently when 90 mEq or more of potassium chloride is administered within 24 hours [12]. To reduce the risk of rebound hyperkalemia, cumulative potassium chloride replacement should generally remain below 90 mEq during the first 24 hours unless there is evidence of ongoing potassium loss [4,12]. When intravenous potassium chloride is required, a practical approach is to administer it at 10 mEq/hour or less, with close cardiac monitoring and serial serum potassium measurements during replacement [4,13].

Potassium supplementation should be individualized and guided by recovery of muscle strength and serial serum potassium measurements [4,13]. Non-selective beta-blockers, such as propranolol, are useful because they reduce beta-adrenergic stimulation and decrease further intracellular potassium shift [11]. Although propranolol can be used as an acute adjunct in TPP, it was initiated in this patient on Day 8, after the documented recurrence of hypokalemia rather than during the initial paralytic episode. Definitive treatment requires control of the hyperthyroid state with antithyroid drugs, radioactive iodine, or surgery, depending on the etiology and clinical context [11].

Several limitations of this case should be acknowledged. As a single-patient report, the findings cannot be generalized to a broader population. Creatine kinase, a spot urinary potassium-to-creatinine ratio, and a calculated transtubular potassium gradient were not available. Although 24-hour urinary electrolytes and urine pH were measured, the collection was obtained after potassium repletion and was not contemporaneous with the blood gas analysis, limiting its usefulness for assessing renal potassium handling and urinary acidification during the initial hypokalemic episode. Consequently, distal renal tubular acidosis could not be definitively excluded. Nevertheless, the overall clinical and biochemical pattern supported TPP as the most likely diagnosis. Genetic testing for familial hypokalemic periodic paralysis was not performed. Finally, although methimazole had been withheld for seven days before thyroid scintigraphy, the potential effect of prior antithyroid therapy on the uptake pattern cannot be excluded. The elevated TRAb level, however, provided independent support for Graves' disease as the underlying etiology.

## Conclusions

This case emphasizes the clinical importance of recognizing TPP as a reversible cause of acute muscle weakness in young patients with hypokalemia and clinical or biochemical evidence of thyrotoxicosis. Although TPP is more frequently described in Asian populations, cases have also been reported in Mexico and Latin America. This case adds to the existing regional literature and illustrates that TPP should be considered in patients with acute muscle weakness, hypokalemia, and thyrotoxicosis regardless of ethnicity.

The complete recovery of muscle strength approximately eight hours after potassium replacement highlights the importance of early identification and timely management. TRAb positivity supported Graves' disease as the underlying etiology of the thyrotoxicosis, while thyroid scintigraphy was interpreted as suggestive of diffuse toxic goiter. During approximately three years of outpatient endocrinology follow-up, no recurrent episodes of TPP were documented, and euthyroidism was achieved within the first year.
